# The mechanism of ferroptosis in early brain injury after subarachnoid hemorrhage

**DOI:** 10.3389/fimmu.2023.1191826

**Published:** 2023-05-17

**Authors:** Xinpeng Deng, Yiwen Wu, Ziliang Hu, Shiyi Wang, Shengjun Zhou, Chenhui Zhou, Xiang Gao, Yi Huang

**Affiliations:** ^1^ Department of Neurosurgery, Ningbo First Hospital, Ningbo Hospital, Zhejiang University, Ningbo, Zhejiang, China; ^2^ Department of Neurosurgery, The First Affiliated Hospital of Ningbo University, Ningbo, Zhejiang, China; ^3^ Cixi Biomedical Research Institute, Wenzhou Medical University, Cixi, Zhejiang, China; ^4^ Medical School of Ningbo University, Ningbo, Zhejiang, China; ^5^ Key Laboratory of Precision Medicine for Atherosclerotic Diseases of Zhejiang Province, Ningbo, Zhejiang, China

**Keywords:** ferroptosis, subarachnoid hemorrhage, early brain injury, oxidative stress, reactive oxygen species

## Abstract

Subarachnoid hemorrhage (SAH) is a cerebrovascular accident with an acute onset, severe disease characteristics, and poor prognosis. Within 72 hours after the occurrence of SAH, a sequence of pathological changes occur in the body including blood-brain barrier breakdown, cerebral edema, and reduced cerebrovascular flow that are defined as early brain injury (EBI), and it has been demonstrated that EBI exhibits an obvious correlation with poor prognosis. Ferroptosis is a novel programmed cell death mode. Ferroptosis is induced by the iron-dependent accumulation of lipid peroxides and reactive oxygen species (ROS). Ferroptosis involves abnormal iron metabolism, glutathione depletion, and lipid peroxidation. Recent study revealed that ferroptosis is involved in EBI and is significantly correlated with poor prognosis. With the gradual realization of the importance of ferroptosis, an increasing number of studies have been conducted to examine this process. This review summarizes the latest work in this field and tracks current research progress. We focused on iron metabolism, lipid metabolism, reduction systems centered on the GSH/GPX4 system, other newly discovered GSH/GPX4-independent antioxidant systems, and their related targets in the context of early brain injury. Additionally, we examined certain ferroptosis regulatory mechanisms that have been studied in other fields but not in SAH. A link between death and oxidative stress has been described. Additionally, we highlight the future research direction of ferroptosis in EBI of SAH, and this provides new ideas for follow-up research.

## 1 Introduction

In 2012, Dixon et al. observed that erastin-induced cell death exhibits a distinct series of morphological, biochemical, and genetic features. This form of death is highly dependent on Fe^2+^, and the accumulation of reactive oxygen species (ROS) and lipid peroxidation (LPO) products is one of the salient features. This process was termed “ferroptosis” (1). Ferroptosis has received widespread interest due to its involvement in development, immunity, aging, and various pathological conditions. Numerous studies have reported that ferroptosis is widely present in multiple diseases such as renal failure, cardiomyopathy, liver cancer, cerebral hemorrhage, stroke, and neurodegeneration (2). After rupture of intracranial blood vessels, the blood enters the subarachnoid space, and this is referred to as subarachnoid hemorrhage (SAH). Within 72 hours after SAH occurs, a sequence of pathological changes occur in the body such as blood brain barrier (BBB) destruction (3, 4), cerebral edema, and neuronal damage that is defined as early brain injury (EBI), and studies have demonstrated that EBI is closely related to poor prognosis. In recent years, researchers and medical professionals have questioned if ferroptosis is involved in early brain injury after SAH. Cao et al. confirmed that ferroptosis is involved in EBI following SAH (2). After SAH, a large number of erythrocytes enter the subarachnoid space and rupture, and the concentration of iron ions increases rapidly (5). Under the mediation of the Fenton reaction using iron as a catalyst, a large number of free radicals such as ROS are generated, and these are a class of molecules that contain partially reduced oxygen such as O_2_^-^, H_2_O_2_, OH^-^, O_3_, and ^1^O2 (6–8). Additionally, under the action of lipoxygenase (LOX), membrane phospholipids containing polyunsaturated fatty acids are directly oxidized to lipid hydroperoxides, and excessive accumulation of reactive oxygen species and lipid peroxides eventually results in cell ferroptosis (9).

## 2 Iron metabolism

After SAH, the blood flowing into the subarachnoid space carries large amounts of hemoglobin and iron that provides the basis for the formation of LPO (6). Ferroptosis is a form of iron-dependent death. Iron acts as an indispensable inducer of lipid peroxidation and ferroptosis that can result in ROS production via the Fenton reaction. It is also used as a synthetic raw material for lipoxygenase and cytochrome P450 oxidoreductase to produce lipid peroxides (10) that ultimately lead to ferroptosis.

**Figure 1:**
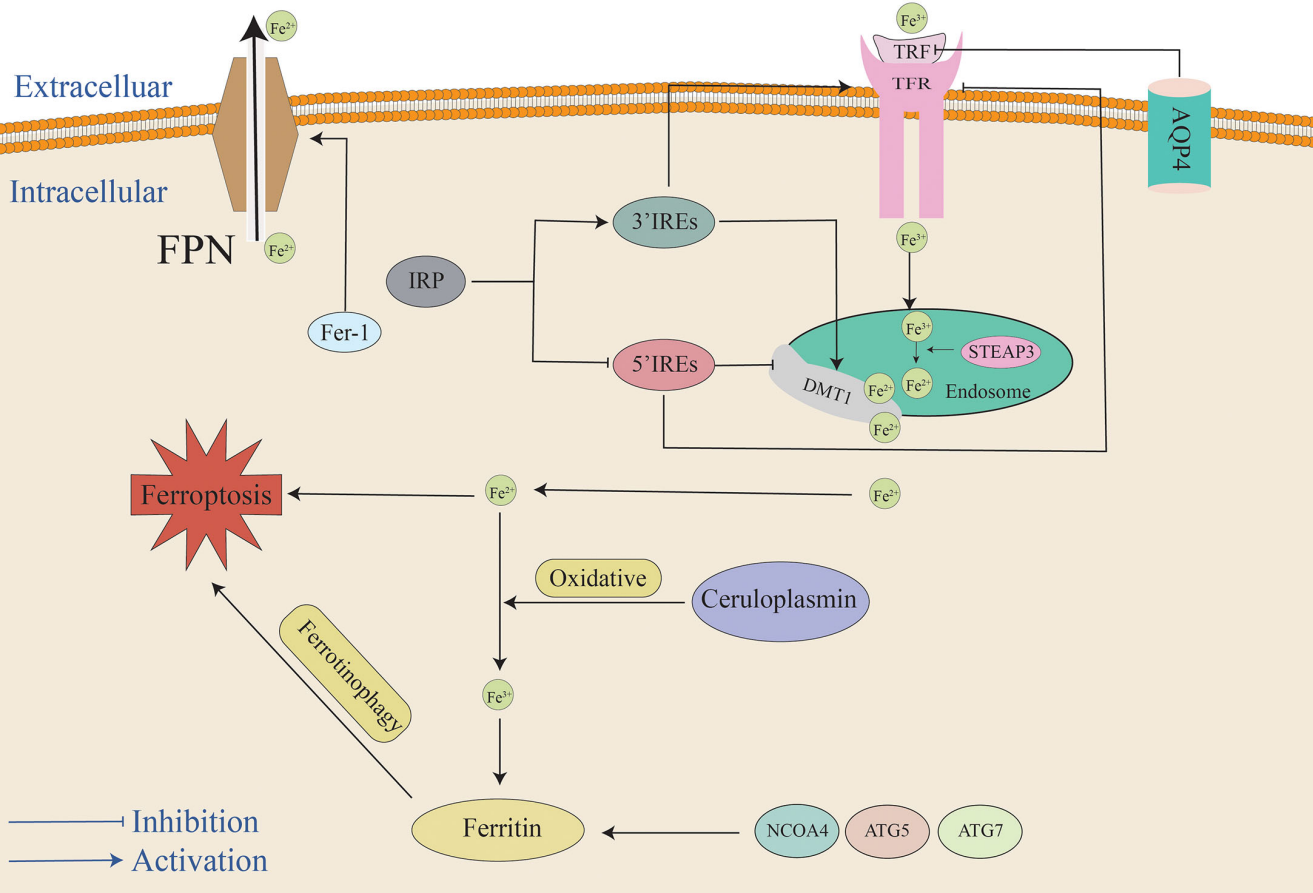
Mechanisms of iron metabolism in ferroptosis. After extracellular Fe3+ binds to TRF, it combines with the TFR to constitute a ternary complex on the surface of the cell membrane. AQP4 can inhibit TRF, and the ternary complex enters the cell and forms the endosome. *In vivo*, transmembrane ferroreductase in endosome reduces Fe^3+^ to Fe^2+^, Fe^2+^ is then transported into cells through DMT1, and IRP regulates the expression of TFR and DMTI by binding to iron response elements at different positions. Fe^2+^ in cells can exist in a free active form or it can be oxidized to Fe3+ by ceruloplasmin to form ferritin. Conversely, ferrotinophagy can also increase intracellular iron content. NCOA4, ATG5, and ATG7 can promote ferrotinophagy. The FPN in the body can transport intracellular iron out of the cell. Elevated intracellular iron levels cause cells to be more susceptible to ferroptosis.: AQP4, aquaporin 4; ATG5, autophagy-related gene 5; ATG7, autophagy-related gene 7; DMT1, divalent metal transporter 1; FPN, ferroportin; Fer-1, ferrostatin-1; IREs, iron-response elements; NCOA4, Nuclear receptor coactivator 4; TFR, transferrin receptors; TRF, transferrin.

Iron homeostasis plays a critical role in the normal life activities of the body, and the body maintains the stability of iron content inside and outside of cells through various metabolic pathways (**Figure 1**). Increasing iron intake or decreasing iron excretion increases cellular susceptibility to ferroptosis. The active iron content in cells is primarily adjusted via the following pathways: 1) ferrotinophagy (11) that is a specific autophagic process that uses ferritin as a substrate; 2) iron uptake mediated by transferrin (12, 13); 3) ferroportin (FPN) that can transfer intracellular iron from cells (14); 4) iron regulatory proteins (IRP) that maintain iron homeostasis by binding to iron response elements in different tissues (15, 16).

Iron is primarily stored and transferred in the form of ferritin complexes that are inert forms of iron that are inactive and cannot promote lipid peroxidation. Ferrotinophagy is an autophagic cell death pathway that uses ferritin as a substrate for its degradation (11). Ferritin consists of a ferritin light chain (FTL) and ferritin heavy chain (FTH). Both FTL and FTH are key indicators of cellular iron homeostasis. The decrease in FTH1 levels marks a decrease in ferritin in the inert form and an increase in active cell-free iron. Abundant ferritin is a key factor controlling ferroptosis sensitivity, and iron is released into unstable iron pools after ferrotinophagy, ultimately resulting in cells that are more sensitive to ferroptosis (17). Nuclear receptor coactivator 4 (NCOA4) is a ferrotinophagy-specific receptor that induces ferritin transfer to autophagosomes and ferrotinophagy (18). Autophagy-related gene 5 (ATG5) and autophagy-related gene 7 (ATG7) mediate ferroptosis by promoting ferrotinophagy, ultimately facilitating increased intracellular iron content and lipid peroxidation (19–21). Ferrotinophagy participates in the pathological process of EBI after SAH. In a study by Liang et al. (11), it was reported that when SAH occurs, ferrotinophagy is accompanied by decreased FTH1 and decreased ferritin in the inert form, and active cell-free iron was increased, eventually leading to iron death. After inhibiting the expression of ATG5, ferrotinophagy was inhibited, the concentration of active iron decreased, and LPO was decreased. Concurrently, ferroptosis-protecting protein content was observed. For example, there is an increase in the expression of glutathione peroxidase 4 (GPX4), and this in turn alleviates ferroptosis induced by SAH and improves the prognostic indicators of SAH. Additionally, studies examining hemorrhagic stroke have demonstrated that the degradation of ferritin and the increase in iron content for various reasons are key causes of brain damage and that the iron chelator desoxamine can alleviate brain damage, thus suggesting that iron overload is an important trigger factor of ferroptosis and providing new insights into the neuroprotective effect of iron chelators (22). These studies also provide a basis for further research focused on ferroptosis in the context of EBI. This is expected to improve the degree of ferroptosis in SAH by regulating ferrotinophagy. These studies not only suggest that SAH causes neuronal ferroptosis by activating ferrotinophagy but also suggest that regulating ferrotinophagy and maintaining iron homeostasis may provide clues for the prevention of EBI (11). It is worth mentioning that autophagy can also mediate the production of lysosomal ROS and can increase the susceptibility of cells to ferroptosis (23, 24). Overall, ferrotinophagy mediates ferroptosis and is anticipated to become a new breakthrough point for the clinical treatment of EBI after SAH.

With the occurrence of SAH, many erythrocytes enter the subarachnoid region and the concentration of extracellular iron ions increases rapidly. Extracellular iron is primarily composed of Fe^3+^ ions. First, Fe^3+^ must bind to transferrin (TRF) and then bind to transferrin receptors (TFR) to form a ternary complex that transports Fe^3+^ into cells across the membrane. Fe^3+^ entering cells form endosomes. Six-Transmembrane Epithelial Antigen of Prostate 3 (STEAP3) reduces Fe^3+^ to Fe^2+^ in endosomes. Fe^2+^ is transported into cells through divalent metal transporter 1 (DMT1, also called SLC11A2), whereas transferrin and transferrin receptors are transported out of the cell. The change in the iron valence is also beneficial in regard to improving the absorption efficiency of iron ions by cells. A portion of the Fe^2+^ entering the cells is oxidized by ceruloplasmin to Fe^3+^ that combines with apoferritin to form ferritin. It becomes inactive storage iron, and the remaining iron enters the cells as Fe^2+^ (25–28). In a rat model of SAH, it has also been reported that the TFR content is significantly upregulated at 24 h after SAH (29). Yuan et al. also observed that ferritin, TFR, and DMT1 levels increased at 6 h in EBI (30). Zhang et al. reported that the iron metabolism-related proteins hepcidin and DMT1 were upregulated in EBI after SAH. After treatment with the DMT1 inhibitor ebselen, the intracellular iron ion concentration decreased, and the degree of ferroptosis was alleviated. These results indicate that ebselen can inhibit EBI by inhibiting DMT1 to decrease intracellular iron content during this period, and this effectively inhibits ferroptosis (31). Taken together, we speculated that SAH induces the upregulation of iron absorption proteins, thus leading to the accumulation of intracellular iron that in turn promotes ferroptosis.

There are not only iron ion transfer pathways in cells but also iron ion excretion channels. Intracellular iron could also be transported out of the cell through transferrin (FPN) that is the sole known iron exporter that regulates mammalian iron export outside of the cell. Contrary to DMT1 playing a role in increasing intracellular iron content, FPN is an important transporter for reducing intracellular iron content (14). Previous studies have revealed that hepcidin is a regulator of iron metabolism. It induces FPN internalization and degradation by combining with FPN (32) and can also increase the expression of DMT1. Therefore, intracellular iron ions become increased. Zhang et al. observed that the iron metabolism-related proteins hepcidin and DMT1 are upregulated and that FPN and GPX4 are reduced in EBI after SAH, and this ultimately causes lipid peroxidation and ferroptosis (31). A study by Li et al. revealed the content of TFR significantly increased at 24 h after SAH, thus resulting in increased intracellular iron concentration, and they also demonstrated that Ferrostatin-1 (Fer-1) treatment could up-regulate FPN expression, reduce iron levels, reduce lipid peroxidation, inhibit the occurrence of ferroptosis, and improve neurological function (29).

IRP is indispensable for maintaining iron homeostasis. It regulates the gene expression of iron-metabolism-related proteins by binding to RNA stem-loop structures that are known as iron-response elements (IREs) that are present in target mRNAs. By combining with IREs at different sites, IRP can regulate iron storage and export, thereby regulating intracellular iron concentration and maintaining intracellular iron homeostasis. If IRP combines with IRE at the 3’UTR of target mRNAs, the expression of TFR and DMTI increases and the intracellular iron concentration increases, whereas if it binds to the 5’UTR of target mRNAs, it will reduce the intracellular iron ion concentration (16, 33). The functions of IRP in the context of ferroptosis have been confirmed in liver cancer studies. α-enolase 1 (ENO1) is an important glycolytic enzyme. Studies have demonstrated that ENO1 inhibits ferroptosis by degrading the mRNA of IRP1 in cancer cells (34). In a study examining melanoma, after treatment with RSL3 and erastin the expression of RP1 was significantly increased, and this increased the TFRC content and inhibited the expression of FPN and FTH1. It increases the level of intracellular iron and promotes ferroptosis. When IRP1 is deficient, intracellular iron accumulation is inhibited and cells are less sensitive to ferroptosis (35). Unfortunately, there have been no studies examining the involvement of IRP in ferroptosis after SAH. We believe that IRP may play a significant role in ferroptosis after SAH; however, this requires further verification through follow-up studies.

Along with the primary regulatory routes for iron metabolism that were already mentioned, aquaporin 4 (AQP4) is among the most abundantly expressed aquaporins in the brain. Under physiological conditions, AQP4 is densely expressed in the form of “polar expression” on the endfoot membrane of astrocytes at the junction of the brain parenchyma and cerebrospinal fluid/blood, and it participates in the formation of the glial limiting membrane that exerts a significant impact on maintaining the dynamic water balance in the brain (13, 36). Further research observed that AQP4 exists in the form of orthogonal arrays of particles (OAPs) on the endfoot membrane of astrocytes and that OAPs are the structural basis for AQP4 to perform its efficient water transport function. Under physiological conditions, AQP4 is primarily located in the membranes of astrocyte end-foot membranes. It is closely related to water transport and is essential for preserving the balance of water and electrolytes between the blood-brain/blood-cerebrospinal fluid; however, under pathological conditions such as AQP4 polarity expression disorder, the formation of OAPs is significantly reduced, the efficient water transport function of AQP4 is impaired, and the water balance between the blood brain/blood cerebrospinal fluid is disturbed, ultimately disturbing the internal environment (13, 36). The study observed that within minutes of SAH, blood components quickly entered the subarachnoid area. Destruction of AQP4 polarization in astrocyte foot processes has been demonstrated to be associated with brain edema (37–39). After SAH, the polarization of astrocyte AQP4 was destroyed, and AQP4 was knocked out. This can aggravate brain damage in EBI by causing brain edema, blood-brain barrier disruption, and neuronal death (40–42). Liu et al. reported that AQP4 also participates in ferroptosis. One potential reason for neuronal ferroptosis is the infiltration of transferrin into the brain parenchyma in EBI after SAH. Overexpression of AQP4 can effectively ameliorate AQP4 polarity loss caused by transferrin infiltration and SAH, thus inhibiting ferroptosis and improving disease prognosis.

## 3 Lipid metabolism and lipid peroxidation

Lipid peroxidation (LPO) is the oxidative deterioration of polyunsaturated fatty acids and lipids. Cell membranes, lipoproteins, and other lipid-containing structures would suffer substantial harm as a result of LPO. LPO can alter the permeability and fluidity of cell membranes, damage DNA and proteins, and affect the normal function of cells, ultimately leading to neuronal death. LPO and anti-oxidation have crucial functions in the metabolic processes occurring within the body. Under normal circumstances, both are in a dynamic balance and maintain the normal progress of many physiological, biochemical, and immune responses in the body. Once this coordination and homeostasis is disturbed and unbalanced, it causes a series of metabolic disorders and decreases immune function, ultimately forming a chain reaction of oxygen free radicals that results in ferroptosis (43).

As a member of the acyl-CoA synthetase long-chain family, acyl-CoA synthetase long-chain family member 4 (ACSL4) is an essential enzyme in fatty acid metabolism. ACSL4 is predominantly expressed in steroid-producing tissues, particularly in the adrenal glands and ovaries. Human ACSL includes ACSL1, ACSL3, ACSL4, ACSL5, and ACSL6, all of which participate in the formation of acyl-CoA from fatty acids (44–46). Although acyl-CoA synthetase long-chain family member 3 (ACSL3) is thought to exert no obvious effect on ferroptosis, in a tumor-related study it was demonstrated that ACSL3-mediated production of monounsaturated fatty acids (MUFAs) limits the oxidation of polyunsaturated fatty acids (PUFAs) and thus inhibits ferroptosis (47), and this also suggests that ACSL3 and ACSL4 may antagonize ferroptosis. Under the action of ACSL4, acyl groups are inserted into PUFAs, and Lysophosphatidylcholine Acyltransferase 3 (LPCAT3) inserts acylated fatty acids into membrane phospholipids. It has been confirmed that phosphatidylethanolamine (PE) containing arachidonic acid (AA) or its derivative epinephrine is a crucial phospholipid that induces cellular lipid peroxidation and ferroptosis (48). In a study by Qu et al., the SAH rat model was used to explore the expression and function of ACSL4 in EBI. This study confirmed that the expression of ACSL4 significantly increased in the brain tissue after brain injury in the early period of SAH. Additionally, they observed that ACSL4 exerted a significant impact on the induction of ferroptosis. Small interfering RNA-mediated inhibition of ACSL4 expression reduces inflammation, BBB damage, oxidative stress, brain edema, and behavioral and cognitive deficits after SAH and increases the number of surviving neurons. They speculated that ACSL4 may cause ferroptosis by mediating lipid metabolism and aggravating brain damage. Additionally, their results revealed that ACSL4 may be utilized as a critical indicator for predicting cell ferroptosis. Reducing the expression of ACSL4 and LPCAT3 is expected to inhibit intracellular lipid peroxide accumulation, and this in turn can inhibit the development of ferroptosis.

The body primarily mediates lipid peroxidation through two pathways after SAH. Additionally, it is worth mentioning that compared to MUFAs, polyunsaturated fatty acid-containing phospholipids (PUFA-PLs) may be a major substrate of lipid peroxidation in ferroptosis in tissues that are thought to be more prone to ferroptosis. The first pathway leading to lipid peroxidation is the non-enzymatic pathway, and this is followed by the enzymatic pathway. Non-enzymatic lipid peroxidation is a free radical-driven chain reaction mediated by the Fenton reaction (49). The Fenton reaction occurs between hydrogen peroxide and Fe^2+^. It is the primary source of reactive oxygen species (ROS) such as the hydroxyl radical (OH −). OH- is one of the most typical chemical forms of ROS and is a highly flexible water-soluble form of ROS that initiates the oxidation of PUFAs (50, 51). As the first step in a non-enzymatic lipid peroxidation reaction, a diene is removed from the acyl moiety of PUFAs in the PUFA-PLs of the lipid bilayers under the action of OH-. This can result in the generation of a carbon-centered phospholipid radical (PL•) that subsequently reacts with an oxygen molecule to form a phospholipid peroxyl radical (PLOO•). It can remove hydrogen from other PUFA to form phospholipid hydroperoxides (PLOOHs) or lipid hydrogen peroxides and new PL•. Without GPX4, they can be converted into the corresponding alcohols (PLOHs). Lipid radicals, specifically PLOO•, PLO•, and PLOOHs, react with PUFA-PLs by removing hydrogen atoms and reacting with molecular oxygen, and this leads to the generation of new PLOOHs and lipid peroxidation (49, 50). As a second pathway mediating lipid peroxidation, enzyme-catalyzed lipid peroxidation is regulated by the activity of a family of lipoxygenases (LOXs). LOXs are a class of non-heme iron-containing enzymes that catalyze the production of numerous lipid hydroperoxides from PUFAs, and of these, arachidonic acid lipoxygenase 15 (ALOX15) plays a major role. Gao et al. reported that cepharanthine (CEP) could reduce EBI after SAH in mice by inhibiting ALOX15-mediated ferroptosis of microglia and endothelial cells (10). Tuo et al. observed that ALOX15 inhibitor can minimize the infarct size following ischemic stroke in a mouse middle cerebral artery occlusion (MCAO) model (52). In mouse ischemic and hemorrhagic stroke treatment models, targeted inhibition of ALOX15 has been observed to exhibit important neuroprotective functions (53). In related studies examining melanoma, it was reported that P53 can regulate ferroptosis through the P53-SAT1-ALOX15 pathway. SAT1, a transcriptional target of P53, is a crucial rate-limiting enzyme in polyamine catabolism. ALOX15 induces lipid peroxidation and ferroptosis following SAT1 activation (54). However, Angeli et al. observed that the genetic removal of ALOX15 did not prevent ferroptosis in mouse fibroblasts after GPX4 knockout and that it did not alleviate acute ischemic kidney injury and related lethality *in vivo* (55). This suggests that ALOX15 is the only pathway that leads to lipid peroxidation. As an essential factor in lipid peroxidation, it has been demonstrated that cytochrome P450 exerts a vital function in both membrane phospholipid peroxidation and subsequent ferroptosis, and targeted inhibition of POR exhibits therapeutic potential in regard to protecting cells from ferroptosis (56). However, the role of the POR in SAH requires further verification.

**Figure 2:**
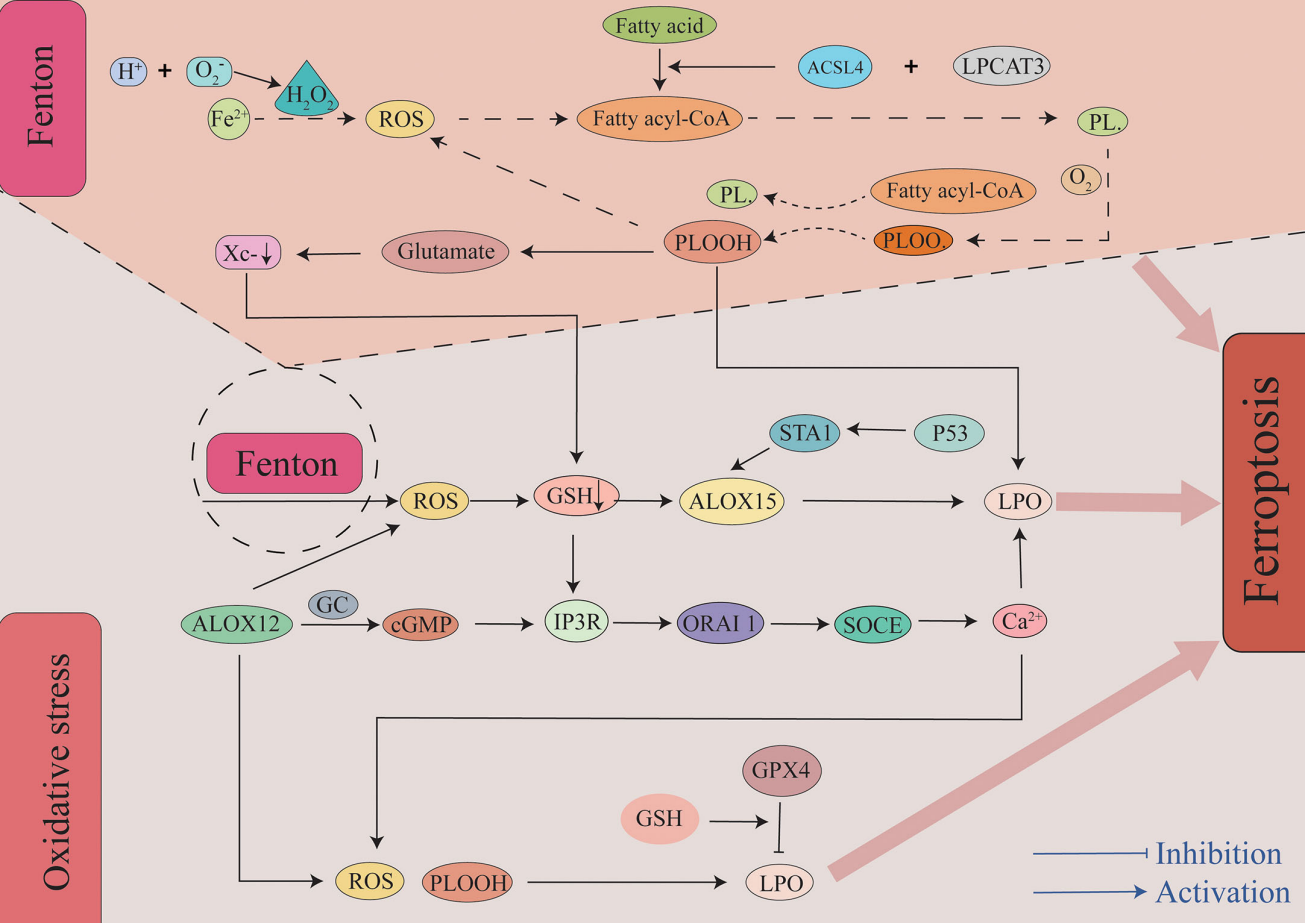
The role of lipid peroxidation in ferroptosis. Insertion of PUFAs into membrane phospholipids under the action of ACSL4 and LPCAT3 causes the lipids to be more susceptible to oxidation. There are two primary pathways leading to lipid peroxidation. First, through a non-enzymatic pathway, the Fe^2+^-mediated Fenton reaction generates a large amount of ROS. A bisallyl hydrogen atom is removed from the PUFA-PLs to form a PL•. It can then react with a molecule of oxygen to constitute a PLOO•. It removes hydrogen from another PUFA to form PLOOH to ultimately lead to the generation of LPO and a new PL•. This forms a vicious cycle that results in a large amount of ROS and LPO. Second, through an enzymatic pathway, ALOX15 oxidizes membrane phospholipids containing PUFAs to generate ROS, and this pathway is also regulated by the P53-STA1 axis. Additionally, metabolite products of ALOX15 and depletion of GSH lead to Ca^2+^ influx, and this in turn leads to the production of ROS. A large amount of ROS and LPO are generated through the above two pathways, and this eventually leads to the occurrence of ferroptosis. ACSL4, acyl-CoA synthetase long-chain family member 4; ALOX15, arachidonic acid lipoxygenase 15; cGMP, cyclic guanosine monophosphate; GC, guanylate cyclase; GPX4, glutathione peroxidase 4; GSH, glutathione; IP3R, inositol triphosphate receptors; LPCAT3, Lysophosphatidylcholine Acyltransferase 3; LPO, lipid peroxidation; ORAI1, calcium release-activated calcium modulator 1; PLOOHs, phospholipid hydroperoxides; ROS, reactive oxygen species; SOCE, store-operated calcium entry.

Large amounts of ROS were produced by enzymatic and non-enzymatic reactions (**Figure 2**). Additionally, many reactive aldehyde by-products are produced such as malondialdehyde (MDA) and 4-hydroxynonenal (4-HNE). Reactive aldehydes such as MDA and 4-HNE can covalently modify biomolecules, including amino lipids and proteins, to produce compounds that can aggravate membrane damage and cause ferroptosis (57). This is precisely due to the toxic effects of lipid peroxides and by-products that occur without converting PLOOH and lipid radicals (especially PLOO• and PLO•) into PLOH by GPX4 that reacts to generate PLOOHs by removing the hydrogen atoms and reacting with oxygen molecules. Ultimately, this chain reaction may destroy the integrity of the cell membrane, ultimately mediating cell death (58).

At the molecular level, lipid peroxides are further decomposed into active substances such as MDA and 4-HNE. They can destroy proteins, lipids, and nucleic acids, ultimately resulting in ferroptosis (59). Structurally, extensive peroxidation of lipids causes biofilm thinning and increased bending and results in further oxidation that ultimately leads to unstable membrane and micelle formation, increased membrane density, significantly constricted mitochondria, shrinking mitochondrial cristae or disappearance, and outer mitochondrial membrane rupture with associated electron-dense characteristics. In contrast, the nuclei of the cells remained structurally intact without condensation or chromatin edges. Ferroptosis occurs under the combined influence of these factors (60). Cao et al. observed the presence of ferroptosis by electron lensing in SAH and observed mitochondrial atrophy, membrane density compression, cristae reduction, and outer membrane rupture (2, 29). Through further quantitative analysis, Li et al. reported that the average mitochondrial area in the SAH group was reduced. However, abnormal changes such as mitochondrial contraction and increased membrane density in the SAH + Fer-1 (ferroptosis inhibitor) group were improved. These studies have further confirmed the existence of ferroptosis in SAH, and the morphological changes in the mitochondria of corresponding cells can be improved by treatment with Fer-1 and other ferroptosis inhibitors (29).

## 4 Antioxidant system

### 4.1 GSH/GPX4 system

**Figure 3:**
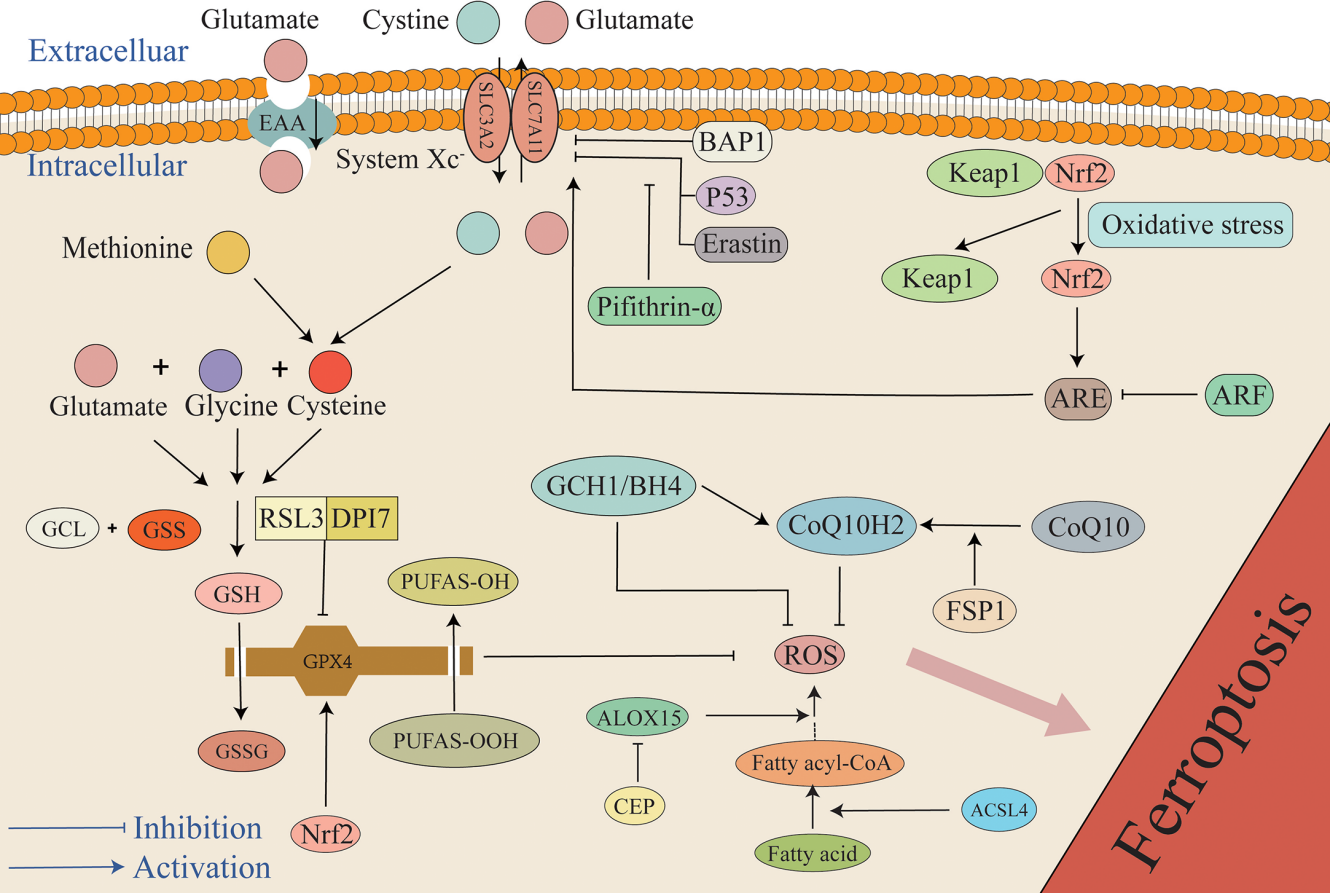
The mechanism of the antioxidant system in ferroptosis. The primary antioxidant system is the GSH/GPX4 system that reduces PUFAs-OOH to non-cytotoxic PUFAs-OH, while GSH is oxidized to GSSG. Under the mediation of the Xc- system, cystine enters cells to synthesize cysteine, and glutamate, cysteine, and glycine synthesize GSH. P53, BAP1, and Erastin can inhibit the antioxidant system by inhibiting SLC7A11. Additionally, pifithrin-α can attenuate the inhibition of SLC7A11 by P53. Under the condition of stress, Keap1 and Nrf2 are separated. Under the mediation of Nrf2, ARE increases cellular resistance to ferroptosis by promoting the expression of SLC7A11, but ARF can attenuate the effect of ARE. Furthermore, RSL3 and DPI7 directly inhibit the antioxidant system by inhibiting GPX4. FSP1 can mediate the conversion of oxidized COQ10 to its reduced form CoQ10H2 that can capture ROS, and it is also regulated by the GCH1/BH4 system. Any target that results in a weakened antioxidant system can result in the overload of lipid peroxides, ultimately causing ferroptosis. ARE, antioxidant response elements; BAP1, BRCA1-associated protein 1; BH4, tetrahydrobiopterin; CEP, cepharanthine; GCH1, GTP cyclohydrolase 1; GCL, glutamate-cysteine ligase; GSS, glutathione synthase; Keap1, Kelch ech-associated protein 1; Nrf2, nuclear factor-erythroid 2 related factor 2.

Although there are various pathways that cause lipid peroxidation and ROS generation, diverse antioxidant systems also exist (**Figure 3**). In the 1950s, Eagle H. et al. confirmed that cysteine is an essential nutrient for many cells, and they observed that cells deprived of cysteine undergo death. The morphology of death differs from that induced by depletion of certain amino acids but possesses a resemblance to the morphology of cell death caused by certain viral infections (61). A study by Bannai et al. further observed that cell death caused by a lack of GSH and cysteine was inhibited by a lipid peroxidation inhibitor (alpha-tocopherol) (62). In 1982, Ursini et al. successfully isolated the enzyme GPX4. As an important antioxidant system, the GSH/GPX4 system is key to cell survival and is the core regulatory protein of ferroptosis. The core mechanism of GPX4 inhibition of lipid peroxidation is the reduction of toxic phospholipid hydroperoxides (PUFAs-OOH) to non-toxic lipid alcohols (PUFAs-OH) in the presence of two molecules of glutathione (GSH) as electron donors, while GSH is oxidized to glutathione disulfide (GSSG) to thereby reduce the accumulation of lipid ROS (63, 64). Wu et al. reported that the induction of ferroptosis by erastin can increase the content of lysosome-associated membrane protein 2a that can promote chaperone-mediated autophagy, thus resulting in the degradation of GPX4 (60, 65). Experiments by Yang et al. demonstrated that RSL3 and DPI7 can directly inhibit the activity of GPX4, thereby causing ferroptosis (66). Liang et al. observed that FIN56 can directly promote GPX4 degradation in tumor-related studies. Additionally, FIN56 combines with squalene synthase, ultimately leading to the exhaustion of endogenous COQ10 to thereby promote ferroptosis (60, 67). Unfortunately, in the EBI after SAH the specific regulation of GPX4 in the process of ferroptosis has not been studied in depth, and it remains unclear if chaperone-mediated autophagy, RSL3, and DP17 participate in the adjustment of GPX4. However, this mechanism requires further investigation.

Gao et al. reported that the content of GPX4 was significantly reduced in rat models of EBI after SAH. Overexpression of GPX4 using adenovirus inhibits lipid peroxidation after SAH *in vitro* and *in vivo*, inhibits ferroptosis, and significantly improves brain edema and neurological dysfunction in rats within 24 h of SAH (68, 69). A study by Li et al. reported that GSH concentration and GPX4 activity were significantly reduced in rat cortical brain tissue after SAH. As expected, the ferroptosis inhibitor Fer-1 effectively increased the content of GSH and GPX4. They also observed that Fer-1 could significantly improve erythrocyte-induced accumulation of ROS, thus suggesting that Fer-1 can prevent ferroptosis in EBI by inhibiting neuronal lipid peroxidation. Additionally, Li et al. used flow cytometry to detect the apoptosis rate of neurons and the caspase-3 protein content. They confirmed that Fer-1can significantly minimize the number of dying neurons, while the number of apoptotic neurons is unaffected. Caspase-3 is an important protein in the apoptotic pathway, and its content was obviously elevated in the Hb and control groups. However, the level of caspase-3 in the Fer-1 group was not reduced, and based on this result, this study suggests that the protective mechanism of Fer-1 in EBI is not related to apoptosis (29). Zhang et al. have demonstrated that the content of GPX4 is significantly decreased in the acute phase of intracerebral hemorrhage and that upregulating the expression of GPX4 could save rats. Additionally, GPX4 is a selenium-containing protein, thus indicating that selenium may be associated with ferroptosis. Actually, it is true that in a rat model of cerebral hemorrhage, selenium supplementation to cells or animals can effectively reduce ferroptosis (70–72). The role of N-acetylcysteine (NAC) is as a precursor of cysteine. In the context of hemorrhagic stroke, studies have demonstrated that NAC treatment exerts an anti-ferroptosis effect through the GPX4-GSH axis, and the toxic effect of heme on primary neurons is significantly eliminated, thus indicating a neuroprotective effect for NAC in the context of hemorrhagic stroke (73). Additionally, NAC can effectively alleviate neuronal cell death and promote functional recovery in rat ICH models by neutralizing lipid peroxidation produced by ALOXs (74). Moreover, the multidrug-resistance pump p-glycoprotein (Pgp) was observed on a genetic screen for controllers of ferroptosis susceptibility. It can pump GSH out of cells, thus resulting in increased sensitivity of MDR1/Pgp-expressing cells to ferroptosis; however, this result has not been confirmed in the SAH model (75). SIRT1 is an epigenetic regulator of gene transcription and affects multiple biological functions such as oxidative stress, inflammation, and mitochondrial biogenesis (76). It has been demonstrated that SIRT1 exhibits strong antioxidant ability and neuroprotective effects in EBI after SAH (77). SIRT1 exerts a strong anti-oxidative ability by decreasing the expression of P53 and NF-kappaB (NF-kB) that can mediate the oxidative stress pathway and by upregulating nuclear factor-erythroid 2 related factor 2 (Nrf2) that mediates the antioxidant stress pathway. Studies have reported that SIRT1 activation can inhibit ferroptosis by increasing the contents of GPX4 and ferroptosis suppressor protein 1 (FSP1) after SAH (30).

Similar to GPX4, copper-zinc superoxide dismutase 1 (SOD1) is an important endogenous enzyme that can eliminate superoxide and is an indispensable peroxidase scavenger in the central nervous system (78). SOD1 overexpression alleviates cell damage following SAH (79). In cerebral ischemia, the neuroprotective effect of SOD1 is partially mediated by activation of serine-threonine kinase (AKT) (80). AKT plays a crucial role in the cell death/survival process (81), and it acts downstream of the phosphoinositide 3-kinase pathway and can function under the action of serine phosphorylation (82). AKT activation promotes cell survival and inhibits apoptosis by phosphorylating and inhibiting downstream substrates, including glycogen synthase kinase 3β (GSK3β). Thus, neurons become resistant to apoptotic stimuli (83). Endo et al. demonstrated that SOD1 overexpression could reduce oxidative stress by activating the AKT/GSK3b survival signaling pathway to thereby attenuate acute brain injury after SAH (84). A study reported that the anti-ferroptosis function of polystyrene nanoparticles is partially dependent upon SOD-mediated ROS scavenging (85). Unfortunately, no studies have confirmed the involvement of SOD in the process of ferroptosis in the context of SAH. This aspect deserves further discussion in subsequent studies.

### 4.2 Xc- system

The antioxidant effect of GPX4 is extremely dependent on GSH, and therefore, the biosynthesis of GSH has also attracted extensive interest. Due to the catalysis of glutamate-cysteine ligase (GCL) and glutathione synthase (GSS), GSH is composed of cysteine, glutamate, and glycine in two stages (86, 87). As raw materials for GSH synthesis, cystine, cysteine, glutamate, and glycine can all affect GSH biosynthesis. Nutrients, including sugars, fats, and amino acids, cannot diffuse directly into cells, and their entry is mediated by specific transporters. Therefore, the components of the amino acids involved in the formation of GSH also require transporters such as the Xc-transporter. The Xc system is a heterodimer transporter formed by a disulfide bond junction that consists of two subunits that include a regulatory subunit composed of solute carrier family 3 member 2 (SLC3A2) and a catalytic subunit composed of solute carrier family 7 member 11 (SLC7A11). The Xc- system promotes the exchange of cystine and glutamate across the cell membrane, where cystine enters the cell and glutamate exits the cell (88). Cystine is reduced to cysteine when transported into the cell. Additionally, another source of cysteine is the reverse transsulfation of methionine (Met) that enters cells through the Xc- system or the ASC system (alanine, serine, and cysteine-preferring) (89). Erastin is a ferroptosis inducer that inhibits Xc expression. Erastin inhibits cystine uptake, thus resulting in the synthesis of the antioxidant GSH that ultimately leads to cell death due to oxidation (90, 91). Furthermore, it has been demonstrated that P53 can affect the expression of the Xc- system by inhibiting the transcription processes required for this system, thus inhibiting the entry of raw materials into cell and resulting in inhibited GSH synthesis. The reduction of GSH in turn results in a weakened antioxidant capacity of GPX4 and greater susceptibility of cells to ferroptosis (92). Studies have reported that intraperitoneal injection of the P53 inhibitor pifithrin-α can increase the levels of SLC7A11 and GSH in rats, reduce lipid peroxidation, reduce neuronal mitochondrial atrophy, and block ferroptosis after cortical SAH, thus indicating that ferroptosis in EBI after SAH depends at least in part on P53 and that P53 plays a role by mediating the Xc- system (particularly SLC7A11). Inhibition of P53 to reduce ferroptosis exhibits the potential to become a new therapeutic target in EBI after SAH (93). It is worth mentioning that not only is P53 a tumor suppressor that participates in the regulation of ferroptosis, but the BRCA1-associated protein 1 (BAP1) tumor suppressor has also been reported to induce ferroptosis by inhibiting SLC7A11 (94).

The concentration of glutamate inside and outside of the cell also exerts an indispensable effect on the Xc- system, and the difference in the concentrations of glutamate and cystine inside and outside the cell drives its own transmembrane diffusion. Glutamate can be continuously transported through its own transporter (EAA) to maintain a high intracellular concentration of glutamate and exported through the Xc- system, thereby supporting the cellular uptake of cysteine (95). Hydrogen peroxide (H_2_O_2_) reacts with Fe^2+^ through the Fenton reaction, ultimately producing a large amount of ROS with the accumulation of glutamate (96). Studies have revealed that a high extracellular glutamate content can not only suppress the function of the Xc- system to result in increased cell sensitivity to ferroptosis but can also be mediated by ionotropic glutamate receptors to lead to Ca^2+^ influx that is cytotoxic (97). Glutamate-mediated oxidative stress toxicity and excitotoxicity are important causes of nerve cell damage in neurodegenerative diseases (1). It has been observed that gastrodin can protect HT-22 cells from glutamate-induced ferroptosis through the Nrf2/HO-1 signaling pathway (98). Clinical studies have demonstrated that excitotoxicity is induced by elevated glutamate concentrations and is associated with cerebral vasospasm and ischemic neurological deficiencies after SAH (99). Sun et al. observed that cerebrospinal fluid (CSF) glutamate levels were significantly elevated within 48 hours after SAH, and ifenprodil improved long-term neurological deficits by antagonizing glutamate-induced excitotoxicity (99). However, further investigation is required to determine if glutamate plays a role in SAH by mediating ferroptosis.

### 4.3 NADPH-FSP1-CoQ10 pathway

CoQ10 is as a crucial component of the mitochondrial electron transport chain that can inhibit lipid peroxidation by trapping free radical intermediates (100). Thus, CoQ10 content plays an indispensable role in the balance of the redox system. CoQ10 depletion renders cells more susceptible to ferroptosis (101). Studies have demonstrated that FSP1 can reduce CoQ10 to its reduced form, CoQ10H2. With the help of NADPH, CoQ10H2 inhibits ferroptosis by trapping lipid peroxy radicals that mediate lipid peroxidation without GPX4 or GSH, and this reveals a novel NADPH-FSP1-CoQ10 pathway that inhibits ferroptosis in parallel with the GPX4/GSH system. Thus, FSP1 is a glutathione-dependent ferroptosis inhibitor (102, 103). Yuan et al. reported that the content of FSP1 and CoQ10 was obviously reduced in both *in vivo* and *in vitro* SAH models, thus suggesting that FSP1-mediated ferroptosis may be involved in EBI after SAH. Additionally, Fer-1 has been demonstrated to increase the content of FSP1, thereby attenuating ferroptosis induced by ferroptosis (30).

Additionally, FSP1 can indirectly influence vitamin E. As a natural antioxidant, vitamin E donates hydrogen atoms to PLOO to form vitamin E radicals (TOC). Immediately thereafter, TOC · can react with other PLOO · to produce a non-radical product, thereby achieving the function of reducing lipid peroxidation products and reducing the seriousness of ferroptosis (104, 105). In a study examining Alzheimer’s disease, when GPX4 was knocked out in specific cerebral cortex and hippocampal neurons, mice exhibited significant cognitive disability in the water maze test and hippocampal neuron degeneration. Ferroptosis has been demonstrated to occur. When mice are fed a diet high in vitamin E, the level of neurodegeneration is reduced, thus indicating that vitamin E confers resistance to ferroptosis (105).

In lung cancer studies, plasma-activated medium induces ferroptosis by depleting FSP1. iFSP1 is considered to be the first FSP1 inhibitor discovered, and ferroptosis can be effectively regulated by targeting FSP1. It has been demonstrated that iFSP1 is able to increase sensitivity to ferroptosis in GPX4-KO cancer cells (102). Overall, these results suggest that the potential of FSP1 in ferroptosis is comparable to that of GPX4. Similarly, in ferroptosis, upregulating FSP1 or stabilizing FSP1 may represent a new direction in regard to improving the poor prognosis of SAH, and this also provides potential therapeutic targets for EBI. As a CoQ10 analog, idebenone stabilizes erythrocyte membranes and reduces lipid peroxidation and the severity of cellular damage in a dose-dependent manner. Idebenone has also exhibited a good therapeutic effect in regard to the treatment of retinal ischemia-reperfusion injury. However, a clinical study examining neuroprotective effects in 57 post-stroke aphasia patients revealed that idebenone did not improve the recovery of brain function compared to that of the placebo group. Thus, treatment with idebenone may possess a narrow therapeutic time window during which it can alleviate damage induced by lipid peroxidation, but it does not inhibit neuronal death after stroke. Therefore, the protective effect of idebenone in the context of stroke must be confirmed by further research, and its effect on the prognosis of SAH requires further study (101, 106).

In addition to the NADPH-FSP1-CoQ10 pathway, new research has identified the GCH1-BH4 pathway that can inhibit ferroptosis without CPX4. This pathway involves the GTP cyclohydrolase 1 (GCH1) gene that is the rate-limiting step in tetrahydrobiopterin (BH4) generation. BH4 inhibits ferroptosis by mediating the production of CoQ10H2 and inhibiting lipid peroxidation. A recent study demonstrated that dihydrofolate reductase (DHFR) can inhibit ferroptosis by regenerating BH4 (107, 108). Kraft et al. observed that the activation of the GCH1/BH4 system can counter lipid peroxidation and alleviate ferroptosis (107).

### 4.4 The ARF/Keap1/Nrf2 pathway

Nrf2 can be activated by dissociation from Kelch ech-associated protein 1 (Keap1) under various stress conditions. Nrf2 recognizes antioxidant response elements (ARE) and activates a series of downstream antioxidant genes. 1) It can up-regulate the expression of GPX4, SLC7A11, and NADPH (109). Gou et al. confirmed that activation of the AKT/Nrf2/GPX4 pathway could alleviate hypoxic-ischemic brain damage (110). Forsythoside A acts against AD by targeting the Nrf2/GPX4 axis to regulate ferroptosis-mediated neuroinflammation. Additionally, inhibition of the Nrf2/GPX4 pathway can activate NF-κB, thus aggravating neuroinflammation (111). 2) The activity of heme oxygenase-1 (HO-1), an inducible enzyme, is important, as HO-1 is considered a measurable indicator of oxidative stress that oxidizes intracellular heme to carbon monoxide (CO), biliverdin, and Fe^2+^ (112). HO-1 exhibits cytoprotective effects by converting pro-oxidative hemoglobin and heme to the antioxidants bilirubin and biliverdin and may also exacerbate oxidative stress by releasing Fe^2+^ and CO. Therefore, HO-1 may exert a dual effect on the regulation of ferroptosis (113, 114). In a study examining retinal epithelial deformation, HO-1 was observed to induce ferroptosis by mediating the Nrf2/SLC7A11/HO-1 axis and the accumulation of ferrous ions (115). Hu et al. reported that β-caryophyllene activated the Nrf2/HO-1 axis to suppress ferroptosis in cerebral ischemia-reperfusion in rats and improve the degree of brain injury (116). Paradoxically, Wei et al. reported in a colorectal cancer study that activating the PERK/Nrf2/HO-1 axis result in ferroptosis (117). Unfortunately, the mechanism of Nrf2/HO-1 in the process of ferroptosis after SAH is currently poorly understood and is worth exploring. 3) NQO1 is a typical Nrf2 target enzyme (118) that exerts a protective effect against ferroptosis (114). NQO1 possesses both superoxide reductase and ubiquitin reductase activities and plays the role of α-tocopherol quinone reductase to convert endogenous α-tocopherol metabolites to the quinoline type, and this is a potent inhibitor of endogenous lipid peroxidation and ferroptosis (119). 4) Nrf2 plays a crucial role in the regulation of iron metabolism genes, including FTH1, FTL, and FPN1 (120, 121). The iron storage protein FTH1 may reduce active iron concentration and inhibit ferroptosis by converting Fe^2+^ to Fe^3+^ (122).

In addition to the Keap1/Nrf2 pathway, recent studies have revealed that the AMPK/PGC1α/Nrf2 pathway plays a role in ferroptosis after SAH. Puerarin is a flavonoid glycoside extracted from Pueraria roots (123). It has been demonstrated that puerarin possesses neuroprotective functions in the context of various central nervous system diseases. As an antioxidant, puerarin maintains the activity of antioxidant enzymes and protects cells from oxidative stress (124, 125) that can induce cell death. It regulates oxidative stress and mitochondrial function via the AMPK/PGC1α/Nrf2 pathways. The activation of this pathway exerts a critical impact on antioxidant activity in the adjustment of oxidative stress and ferroptosis (126). Previous research has demonstrated that in a rat model of hypoxic-ischemic encephalopathy, promoting AMP-activated protein kinase (AMPK) phosphorylation and upregulating PGC1α expression can exert neuroprotective effects by reducing oxidative stress and neuronal apoptosis. As a major antioxidant regulator, Nrf2 is regulated by the AMPK/PGC1α signaling pathway (127, 128). To explore the mechanism of puerarin in SAH, Huang et al. observed that puerarin reduced oxidative stress and ferroptosis after SAH through activating the AMPK/PGC1α/Nrf2 axis and also improved neurobehavioral disorders to a certain extent (129).

As one of the key regulators of antioxidant stress pathways (130), Nrf2 is normally maintained at low levels through ubiquitination mediated by the tumor suppressor Keap1. Glycogen synthase kinase 3β (GSK3β) is the primary negative regulator of Nrf2 activity, and hyperactivation of GSK3β leads to phosphorylation of specific serine residues in the Neh6 domain of Nrf2 to form a phosphorylated domain for degradation, ultimately resulting in Nrf2 inhibition. Studies have demonstrated that the antioxidant effect of Nrf2 is impaired by upregulation of Keap1 and activation of GSK3β (131). Another negative regulator, BTB domain and CNC homologue 1 (BACH1), inhibits the expression of Nrf2 target genes (e.g., HO1, NQO1, and xCT) by competing with Nrf2 to bind to ARE sequences (132). Namgaladze et al. revealed that silencing of BACH1 reduces labile iron pools and lipid peroxidation and enhances macrophage resistance to ferroptosis (133). Nrf2 was also regulated by ARF. ARFdoes not regulate Nrf2 protein content by interfering with Keap1-mediated ubiquitination but instead suppresses CBD-dependent Nrf2 acetylation that inhibits the expression of NRF2. Conversely, ARFdeletion induces Nrf2 activation and increases cellular resistance to ferroptosis. Additionally, certain miRNAs can alter the susceptibility of cells to ferroptosis by regulating the Nrf2 content. As an important inhibitor of ferroptosis, the function of NRF2 also exhibits other functions. It can also inhibit ROS generation to decrease the susceptibility of cells to ferroptosis (130). In a rat model of transient middle cerebral artery occlusion, Nrf2 concentration increased after 2 h, peaked at 8 h, and decreased between 24 and 72 h (134). The Nrf2 concentration is obviously higher in the penumbra than it is in the core (135), and this may be due to higher oxidative stress in the penumbra (134). TBHQ that can activate Nrf2 can improve Nrf2 activity and significantly reduce brain cell death.

## 5 Hippo–YAP pathway

Hippo–YAP signaling participates in various biological functions, including cell proliferation and organ size control (136) and is an important pathway in tumorigenesis and development. In a study examining breast tumors, Wu et al. reported that cells grown at high densities tended to be less sensitive to ferroptosis caused by cysteine depletion and GPX4 suppression. This also provided an opportunity for the discovery of the Hippo-YAP pathway in ferroptosis. Further studies have reported that intercellular interactions lead to ferroptosis in tumor cells by mediating the NF2-YAP pathway and that YAP promotes the transcription of key ferroptosis genes such as ACSL4 and TFRC. They observed that inhibiting the expression of Hippo or promoting the expression of YAP increased the susceptibility of cells to ferroptosis (137). However, paradoxically, a study by Gao et al. that focused on hepatocellular carcinoma reported that YAP/TAZ, as a transcriptional coactivator, formed a complex with TEADs that indirectly bound the TEAD sequence in the SLC7A11 gene promoter, ultimately leading to upregulating the content of SLC7A11 and inhibiting the development of ferroptosis (138). The role of the Hippo-YAP pathway in SAH remains poorly studied.

## 6 Energy stress AMPK pathway

Energy stress depletes ATP and leads to cell death. Additionally, energy stress and glucose starvation increase ROS production (139, 140). Glucose starvation has been speculated to induce ferroptosis. In contrast, glucose starvation was previously demonstrated to effectively suppress ferroptosis (126). This study reported that this protective effect under energy stress is mediated by the activation of AMPK. When glucose starvation occurs, AMPK is activated, and this inhibits the biosynthesis of PUFAs. The lipid peroxidation drive of PUFAs is critical for ferroptosis (48, 141). Exhaustion of intracellular ATP and corresponding improvement in intracellular AMP concentration during energy stress activates AMPK by binding to AMP. Acetyl-CoA carboxylase 1 (ACC1) and Acetyl-CoA carboxylase 2 (ACC2) are two related enzymes that promote the synthesis of malonyl-CoA from acetyl-CoA and possess the functions of promoting fatty acid synthesis. Activated AMPK inhibits ACC1 and ACC2 that mediate fatty acid synthesis under energy stress, thereby leading to resistance to ferroptosis (126).

## 7 Relationship between oxidative stress, lipid peroxidation, and ferroptosis

**Figure 4:**
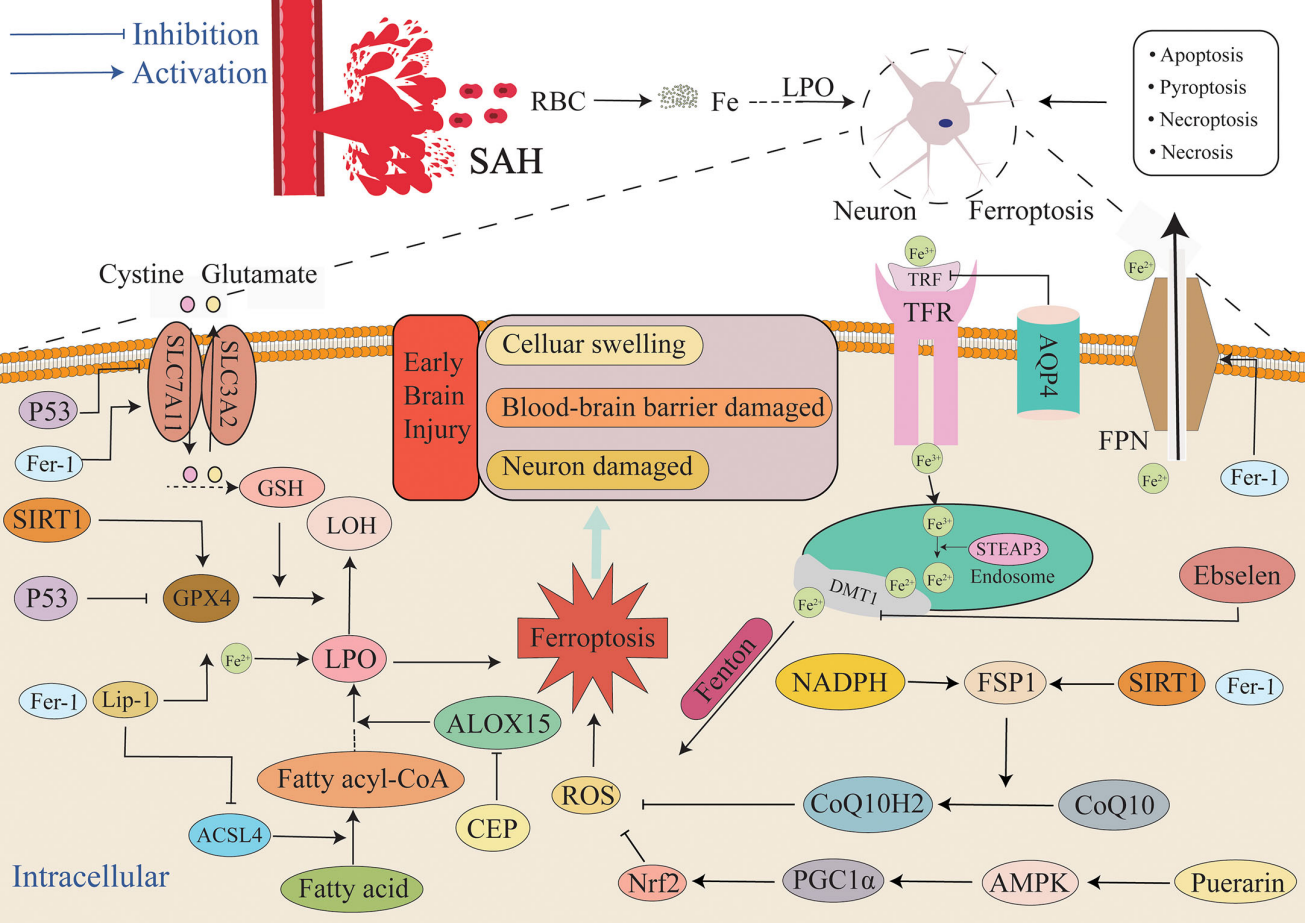
Mechanism of ferroptosis in EBI after SAH. After SAH, a large amount of blood and ruptured erythrocyte flow into the subarachnoid space, and ferroptosis, necrosis, apoptosis, necroptosis and pyroptosis can all lead to the death of neurons and other cells. This article focuses on ferroptosis, an iron-independent cell death mechanism characterized by lipid peroxide accumulation that exacerbates EBI. The extracellular environment is primarily Fe^3+^, and it is primarily combined with TRF and enters into cells through TFR. After entering cells, endosomes are formed, Fe^3+^ is reduced to Fe^2+^ by STEAP3, and Fe^2+^ is diverted into cells via DMT1. Fe^2+^ mediates ROS production by the Fenton reaction. FPN can also reduce the intracellular iron concentration by transporting iron ions out of the cell. Additionally, the GSH/GPX4 and FSP1/COQ10 systems act as the primary antioxidant systems to suppress the production of lipid peroxides, and the imbalance between the oxidation system and the antioxidant system will lead to the accumulation of lipid peroxides and finally lead to ferroptosis. Aquaporin 4 can also reduce iron ion concentration and the severity of ferroptosis by inhibiting TFR. AMPK, AMP-activated protein kinase; DMT1/SLC11A2, divalent metal transporter 1; FSP1, ferroptosis suppressor protein 1; SLC7A11, solute carrier family 7 member 11.

After SAH occurs, the blood components enter the subarachnoid space. Various pathways and oxidative and antioxidant systems regulate the occurrence of ferroptosis (**Figure 4**), and they also regulate neuronal ischemia and hypoxia, mitochondrial dysfunction, and the production of a large amount of ROS during electron transfer (142, 143). ROS induces a local inflammatory response, thus triggering a downstream inflammatory cascade that causes a near-exponential increase in ROS that ultimately leads to the development of oxidative stress. Additionally, the immune system is activated, and many peripheral inflammatory cells enter the subarachnoid region under the chemotaxis of inflammatory cytokines. The inflammatory cells secrete a variety of inflammatory cytokines, thus forming a vicious cycle that results in the generation of numerous ROS (144). Antioxidant systems such as GSH/GPX4 scavenge ROS, and the imbalance between the generation of ROS and the antioxidant system leads to the accumulation of lipid peroxides and ROS (145, 146) that in turn leads to ferroptosis. Ferroptosis exhibits features that include lipid peroxide accumulation and iron dependence, and it is the most likely form of cell death in response to oxidative stress. Superoxide produced by oxidative stress reacts with H^+^ to produce H_2_O_2_. A large number of erythrocytes entering the subarachnoid space after SAH lead to an increase in the concentration of iron ions, and the Fenton reaction between ferrous iron and H_2_O_2_ occurs (96), ultimately resulting in the production of highly active OH- with the accumulation of glutamate. The occurrence of lipid peroxidation mediated by ROS species such as OH- causes the accumulation of more lipid peroxidation, while the high extracellular concentration of glutamate inhibits the Xc- system and results in exhaustion of GSH and inhibition of GPX4. It activates ALOXs that use iron as a cofactor and react with membrane phospholipids containing unsaturated fatty acids to generate large amounts of lipid peroxides that further attack and oxidize cell membrane lipids and trigger ferroptosis (147). Concurrently, GSH is depleted by a large amount of ROS, ultimately resulting in the activation of inositol triphosphate receptors (IP3R) to thereby deplete calcium stores in the endoplasmic reticulum and trigger the activation of calcium release-activated calcium modulator 1 (ORAI1). Activation of store-operated calcium entry (SOCE) then leads to a growth in intracellular Ca^2+^ concentration, and this further leads to the production of a large amount of ROS. It not only aggravates oxidative stress, but also leads to ferroptosis due to excess ROS. The change in Ca^2+^ is not only affected by GSH content, but in turn, the changed Ca^2+^ also further depletes GSH by boosting the generation of ROS. The depletion of GSH then leads to the inactivation of GPX4, ultimately causing the accumulation of lipid peroxides that induce ferroptosis (148). Moreover, ALOXs can not only directly react with lipids to produce lipid hydroperoxides, but the metabolites of 12-LOX can also activate soluble guanylate cyclase (GC) to generate cGMP. Activation of ORAI1 and SOCE by cGMP promotes the influx of Ca^2+^ into cells (149, 150) and causes ROS production. Both oxidative stress and ferroptosis are caused by massive accumulation of oxides. GSH plays a major role in anti-oxidation (151). Therefore, both ferroptosis and oxidative stress result in a decrease in GSH that leads to damage to the related antioxidant system. Finally, the production of ROS is greater than its elimination, and the redox system is unbalanced, thus resulting in cytotoxicity. SIRT1 is a III histone deacetylase that can regulate multiple cellular biological processes such as inflammation, oxidative stress, energy metabolism, DNA damage repair, and cell death (152, 153). There is increasing evidence that neuroinflammation is firmly connected to the pathogenesis of a number of neurological diseases (154). Numerous studies have demonstrated that SIRT1 positively affects neuroinflammation-associated disease. For example, Hernández-Jiménez et al. reported that SIRT1 could reduce cerebral ischemia-induced neuroinflammation and neuronal damage by suppressing P53 and NF-κB acetylation (155). The neuroprotective effect of SIRT1 in the context of cerebral ischemia is mediated by multiple mechanisms. After ischemic stress, DNA damage and oxidative stress activate P53 that in turn promotes mitochondrial apoptosis signaling and neuronal death (156–158). SIRT1 inhibition of P53 can suppress apoptosis, promote cell survival, and protect neurons from ischemia-induced cell death (159, 160). SIRT1 gene deletion or pharmacological inhibition increases peri-infarct area (155). Increasing numbers of studies examining SAH have demonstrated that SIRT1 is extensively expressed in the brain and possesses an endogenous neuroprotective function in EBI through regulating oxidative and inflammatory signaling (76, 161–163). SIRT1 activation improves EBI neural function by inhibiting the inflammatory response to oxidative stress, whereas SIRT1 silencing aggravates SAH-induced brain damage. Yuan et al. up-regulated the content of SIRT1 via RSV pretreatment and decreased the content of SIRT1 via SEL pretreatment. The experimental results demonstrated that the artificial overexpression of SIRT1 through RSV mediated the upregulation of GPX4 and FSP1 expression and significantly reduced the concentration of lipid peroxidation, and this significantly alleviated ferroptosis. Furthermore, inhibition of SIRT1 activation via SEL reduced GPX4 and FSP1 concentrations and induced neuronal ferroptosis. Specifically, this suggests that SIRT1 exerts a neuroprotective effect in the context of ferroptosis when the intracellular antioxidant system is activated (30). In conclusion, SIRT1 not only inhibits oxidative stress to a certain extent but also confers resistance to ferroptosis. In summary, it can be observed that ferroptosis and oxidative stress are very similar in many aspects, and ferroptosis cannot simply be considered as an independent cell death pathway and may even be a more particular form of oxidative stress outcome.

## 8 Crosstalk between microglia, astrocytes and neurons in ferroptosis

After SAH, different cells in the brain play different roles and respond differently with the severity of the disease. Glial cells, comprising astrocytes and microglia, act as vigilant protectors of neurons, working to preserve the integrity of the blood-brain barrier, regulate synaptic activity, and respond to injury within the central nervous system (CNS) (164). Glial cells are known to express a range of iron transporters and iron metabolizing proteins, which are critical for maintaining iron homeostasis and ensuring proper functioning of the brain (165, 166). In order to maintain a balance of iron in the body, it is important for certain cells of the immune system, such as microglia and astrocytes, to play a role. Bind free iron via cytoplasmic and mitochondrial ferritin, thereby reducing extracellular iron concentrations. Astrocyte-neuron interactions protect neurons from iron-mediated cytotoxicity, and circadian regulation of BDNF-mediated Nrf2 activation in astrocytes protects dopaminergic neurons from ferroptosis (167). Microglia are the most sensitive to ferroptosis (168), When microglial iron homeostasis is unbalanced, excessive ROS and inflammation will be produced, accompanied by increased free iron (169), which will cause cytotoxicity to other cells such as neurons, leading to oxidative stress and ferroptosis (170). The development of single-cell sequencing has significantly contributed to our profound comprehension of the intercellular communication during ferroptosis (171, 172). Through single-cell RNA-sequencing, Chen et al. identified multiple SAH-specific microglial cluster (SMG-C) in a mouse model of SAH, among which the corresponding genes for SMG-C5, SMG-C6, and SMG-C7 were only highly expressed in SAH microglia and not in normal microglia. These SMG-C subgroups were closely associated with neuroinflammation, oxidative phosphorylation, and apoptosis after SAH (173, 174). Additionally, Dang et al. also found that ferroptosis was activated in astrocytes and may be involved in the pathological process of Alzheimer’s disease (175). Zhang et al’ study identified ten cell types including cholinergic neurons, dopaminergic neurons, glutaminergic neurons, neuronal precursors, microglia, oligodendrocytes Cells and radial glial cells, etc., and revealed ferroptosis as a new mechanism of manganese-induced neurotoxicity (171). Overall, single-cell RNA sequencing can be used to analyze different types of cells in brain tissue, identify cell types and signaling pathways associated with ferroptosis, and investigate the relationship between gene variations or genomic variations in specific cells and ferroptosis, thereby providing clues for potential therapeutic targets for SAH.

## 9 Detecting indicators of ferroptosis

As ferroptosis is inextricably linked to the EBI of SAH, timely judgment and effective means to inhibit the occurrence of ferroptosis are extremely important in regard to the prognosis of EBI. Ferroptosis can be induced in iron metabolism by detecting intracellular iron content. After a mild traumatic brain injury, iron accumulation in certain regions of the thalamus can indicate the likelihood and severity of future post-traumatic headaches. Specifically, patients who have suffered an acute traumatic brain injury have been found to have higher iron deposition in the left lateral geniculate nucleus compared to healthy controls. This increased iron deposition in the left lateral geniculate nucleus may be indicative of the severity of the injury and could potentially lead to a poorer recovery from post-traumatic headaches (176). Furthermore, research has indicated that serum iron levels at admission can serve as an independent risk factor for delayed cerebral ischemia following SAH (177). The buildup of iron in the brain may be connected to secondary brain injury in individuals with SAH. In patients with poor grade SAH, iron accumulation is often observed in the white matter. Higher levels of intraventricular hemorrhage are correlated with higher levels of iron deposition. Additionally, patients with vasospasm have been found to have higher levels of iron compared to those without vasospasm (176). TFR plays an important role in intracellular iron metabolism. It has been demonstrated that the expression of TFR is significantly positively correlated with the severity of ferroptosis and that apoptosis is not related. Therefore, the TFR is considered as a marker of ferroptosis (178). Ferritin also plays a crucial role in iron homeostasis. FTH1, an important component of cellular ferritin, is a biomarker that reflects intracellular iron homeostasis. In general, higher levels of FTH1 cause cells to be more resistant to ferroptosis (11, 178). This is due to ferroptosis being a result of lipid peroxidation. As an upstream molecule of lipid peroxidation, it has been demonstrated that the expression of ACSL4 is up-regulated, while the expression of other ACSL family members is not upregulated. ACSL4 is a biomarker that predicts ferroptosis sensitivity (179). Additionally, ROS, lipid peroxides, and other oxidation products are significantly correlated with ferroptosis. Metabolites of lipid peroxides such as malondialdehyde (MDA) and 4-HNE are crucial markers of the severity of ferroptosis. Within 2 h after stroke induction, the levels of 4-HNE in the ischemic cerebral cortex were increased (180). Lee et al. further demonstrated that plasma 4-HNE concentration increased in ischemic stroke and became a potential biochemical indicator of ischemic stroke (180, 181). The ability of GPX4 to convert toxic PUFAs-OOH to non-toxic PUFAs-OH in the presence of GSH such as GPX4 and GSH is often considered to be highly correlated with ferroptosis markers. The higher the content of GPX4 and GSH, the less prone they are to ferroptosis (63, 64). However, these indicators are non-specific and can be influenced by many other diseases such as oxidative stress.

## 10 Outlook and conclusion

Compared to other types of stroke, SAH is a life-threatening cerebrovascular disease that seriously affects the quality of life (182). Although the morbidity and mortality of SAH have declined due to emerging therapies and improvements in clinical management, both remain high (183). As the focus of research has shifted from cerebral vasospasm to EBI, it has been observed that the role of ferroptosis in SAH is particularly important. Experimental and clinical data have demonstrated that ferroptosis is effectively inhibited by regulating iron metabolism, lipid peroxidation, and the CPX4 and Xc systems, and the adverse EBI outcome is improved (2, 10, 11, 29, 31, 68, 93, 184). It has been demonstrated that there are multiple forms of cell death in the context of EBI (185). For example, ferroptosis, necrosis, apoptosis, necroptosis, and pyroptosis that ultimately lead to the poor prognosis of SAH all occur. Therefore, there is crosstalk between ferroptosis and other forms of death that can be synergistic or antagonistic and occur as upstream or downstream reactions. This series of questions is worthy of further investigation. After SAH, the iron ion is one of the initiating factors of ferroptosis, and changes in its concentration are closely related to ferroptosis. Previous studies examining SAH have primarily focused on iron-related transporters and ferrotinophagy. However, IRP is an important target for iron concentration regulation (186, 187), and its role in ferroptosis after SAH has rarely been studied. We speculate that IRP as an important regulatory factor in iron metabolism may exert an indispensable effect on ferroptosis in the context of EBI. Research has revealed that the influx of Ca^2+^ also leads to the generation of ROS (149). Therefore, other inorganic ions such as calcium ions are involved in ferroptosis. An imbalance between the oxidative and antioxidant systems that causes the collection of ROS is the primary cause of ferroptosis. Studies have demonstrated that the GSH/GPX4 system is the primary antioxidant system, and thus, it is likely that the SOD antioxidant and catalase systems are involved in SAH. Little research has been conducted examining the role of SOD in ferroptosis in EBI. It remains unclear if it can become a new therapeutic target in SAH and improve the lethality and mortality of clinical patients. Currently, in related research examining ferroptosis after SAH, the primary research focuses on the GSH/GPX4, NADPH/FSP1/COQ, and other pathways; however, the Hippo/YAP pathway has exhibited good anti-ferroptosis in tumor-related research (136, 137, 188). It has not yet been verified if it is involved in ferroptosis after SAH. If it is involved, it is worth exploring if the pathway depends upon the GSH/GPX4 pathway. This review provides a plausible conjecture regarding the interrelationship between oxidative stress and ferroptosis in EBI after SAH, but there is growing evidence that there are also interactions between ferroptosis and other types of cell death. It is necessary to fully explore the relationship between various forms of death and ferroptosis and to determine if there is a common pathway. Answering these questions may provide a new therapeutic target for SAH, ultimately improving the poor prognosis of the majority of SAH patients and reducing family and social burden. However, research focused on ferroptosis still faces challenges, as we do not fully understand the mechanisms related to ferroptosis in the context of SAH.

## Author contributions

YH and XG contributed to the conception and design of the study. XD, YW, ZH, SW, SZ and CZ organized the database. XD wrote the first draft of the manuscript. YH reviewed and edited. All authors contributed to the article and approved the submitted version.
